# Bevacizumab-refractory radiation necrosis with pathologic transformation of benign meningioma following adjuvant gamma knife radiosurgery: A rare case report

**DOI:** 10.1097/MD.0000000000021637

**Published:** 2020-07-31

**Authors:** You-Sub Kim, Woo-Youl Jang, Kyung-Hwa Lee, Kyung-Sub Moon, Tae-Young Jung, Shin Jung

**Affiliations:** a Department of Neurosurgery; b Department of Pathology, Chonnam National University Hwasun Hospital and Medical School, Hwasun, Jeollanam-do, South Korea.

**Keywords:** radiation necrosis, peritumoral edema, bevacizumab, transformation

## Abstract

**Rationale:**

Bevacizumab has shown good efficacy in radiation necrosis (RN) following gamma knife radiosurgery (GKRS) and associated peritumoral edema. However, few studies have reported bevacizumab failure. Moreover, the pathologic transformation of benign meningioma following GKRS has never been reported.

**Patients concerns:**

A 41-year-old man was admitted with focal seizure on the right arm.

**Diagnoses:**

Magnetic resonance imaging (MRI) demonstrated a 4.7 cm-sized convexity meningioma involving left motor cortex.

**Interventions:**

Subtotally resected tumor was confirmed as a meningothelial meningioma and subsequently treated by GKRS. During 4-year follow-up after GKRS, seizure and hemiparesis had persisted with progressively worsened peritumoral edema regardless of steroid and bevacizumab treatment. Radical debulking of tumor was achieved and immunohistopathological examination revealed angiomatous meningioma with necrotic core presenting scanty VEGF expression.

**Outcomes:**

A follow-up MRI at 4 months after debulking surgery showed a marked reduction of peritumoral edema with improvement of symptoms.

**Lessons:**

This is the first report of pathologically confirmed angiomatous transformation following GKRS. Although the pathogenesis is not fully understood, this rare pathologic transformation may be closely related to RN. Also, if bevacizumab is resistant, debulking surgery for reducing tumor burden could be an effective treatment option to control the RN.

## 1 Introduction

Gamma knife radiosurgery (GKRS) has been widely used as a primary or adjuvant treatment for intracranial meningiomas. The consensus is that GKRS is an effective treatment modality irrespective of whether primary or adjuvant treatment, with long-term tumor control rate of >80%.^[1–3]^

Radiation necrosis (RN) is one of the most common complications following GKRS accounting for 10% of patients.^[4]^ It is often accompanied by peritumoral edema resulting in progressive neurologic deficits. Vascular endothelial growth factor (VEGF) has been generally accepted as a key factor for RN.^[5–7]^ VEFG, a known regulator of angiogenesis and vascular permeability, is expressed in both necrotic core and peritumoral brain tissue.^[8]^ Bevacizumab, an anti-VEGF antibody, has shown definite efficacy to RN.^[9,10]^ It is more specific to RN than other medical treatments with evidence of radiological and clinical improvement. However, most studies have only focused on bevacizumab efficacy during short follow-up period without pathologic confirmation and no studies have reported bevacizumab resistance.

More interestingly, pathologic transformation of benign meningioma has never been reported. Only one case of angiomatous lesion was reported, but it lacked evidence because pathologic evaluation was not performed before GKRS.^[11]^ Thus, it is not clear whether angiomatous lesion occurred after GKRS.

In this report, we present the case of a patient with bevacizumab-refractory RN following adjuvant GKRS for benign meningioma and discuss the possible relation with this refractory RN and pathological angiomatous transformation.

## 2 Case Presentation

Written informed consent was obtained from the patient for publication of this case report and accompanying images

### 2.1 History

**Figure 1:**
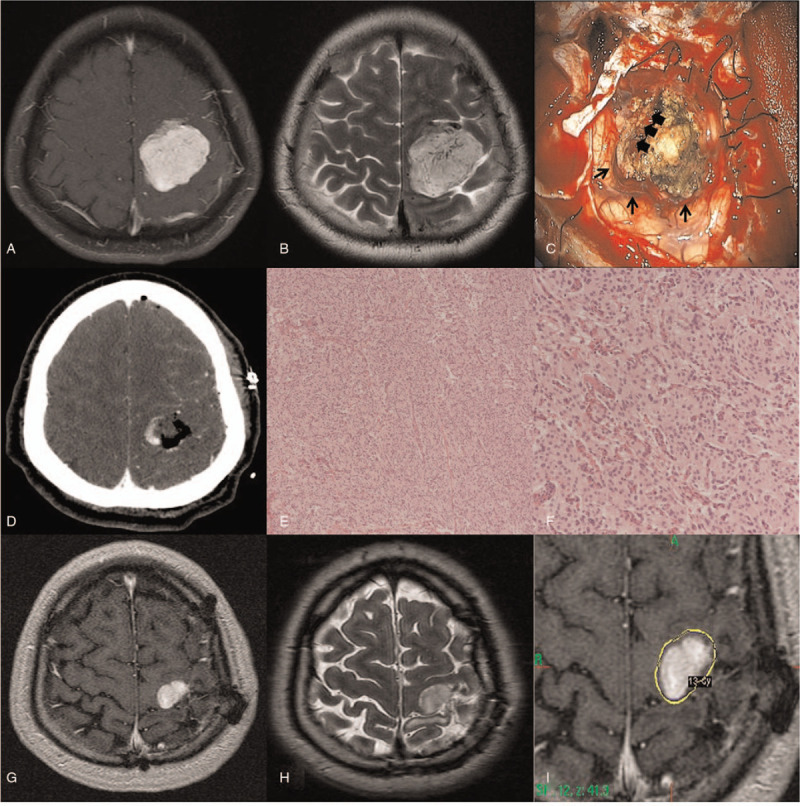
Contrast-enhanced brain MRI showed a 4.7 cm-sized heterogeneously enhanced mass in left motor cortex with minimal peritumoral edema. (A and B) Due to the severe adhesion and rich cortical veins around the tumor, subtotal resection was achieved. (C and D) Photomicrograph demonstrated densely packed cells arranged in sheets with no clear cytoplasmic borders, indicating meningothelial meningioma. (E and F) MRI at 10 months after first operation revealed regrowth of tumor, so GKRS was performed with marginal dose of 13 Gy at 50% isodose line. (G–I) Arrows indicate rich cortical veins around tumor, and arrowheads indicate severe adhesion of tumor to the cortex.

A 41-year-old man presented with focal seizure on the right arm. Contrast-enhanced magnetic resonance imaging (MRI) revealed an ∼4.7 cm sized, well defined, and heterogenously enhanced mass with minimal edema in the left motor cortex, consistent with a convexity meningioma. A left frontoparietal craniotomy was performed and the tumor was subtotally resected because significant cortical adhesion with rich cortical veins around tumor was observed. There was no decrease or change on intraoperative neurophysiologic monitoring. Microscopically, the tumor was confirmed as a meningothelial meningioma (WHO grade I) without necrosis. Regrowth of the remnant tumor was observed at 10 months after surgery, so GKRS was performed with marginal dose of 13 Gy at 50% isodose line (Fig. 1).

### 2.2 Clinical course, pathologic findings, and postoperative course

**Figure 2:**
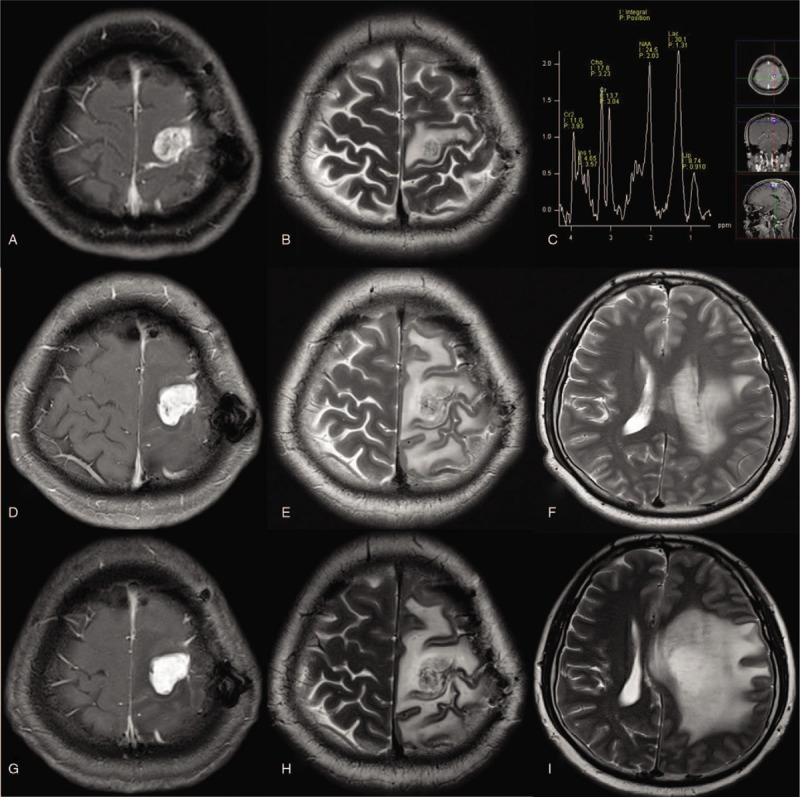
MRI at 3 months after GKRS demonstrated increased size of the tumor and peritumoral edema with lactate peak suggesting radiation necrosis. (A–C) Serial follow-up MRIs obtained at 9 months (D–F) and 18 months (G–I) after GKRS shoed progressively worsened peritumoral edema with midline shifting regardless of steroid treatment.

After 3 months of GKRS, focal seizure recurred and MRI revealed RN with slightly increased edema. At first, the seizure was well controlled with steroid and antiepileptics. On follow-up MRI 9 months after GKRS, however, significantly increased peritumoral edema was observed. Subsequently, focal seizure had persisted once to twice a week with hemiparesis of motor grade 4-/4-strength. It was difficult to taper the steroid and antiepileptics due to the progressively worsening hemiparesis with repeated seizure. A follow-up MRI at 18 months after GKRS demonstrated sustained severe peritumoral edema (Fig. 2).

**Figure 3:**
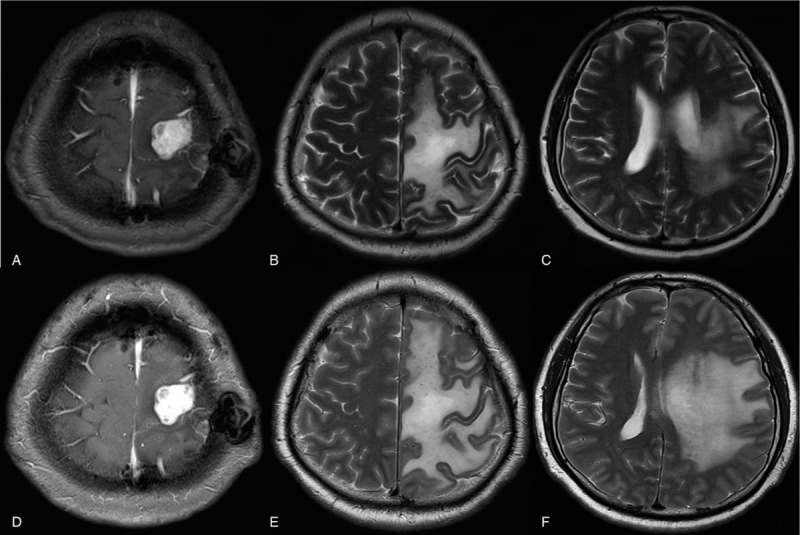
There was a slight decrease in T2 high signal intensity on MRI after 8th cycle of bevacizumab (A–C). However, peritumoral edema was worsened again on MRI despite additional 4 cycles of bevacizumab (D–F).

So, 8 cycles of bevacizumab were planned (5 mg/kg every 4 weeks for 8 months). The response and efficacy of the bevacizumab was determined by both radiologic improvement (decrease edema in T2-weighted MRI) and clinical improvement. After administration of 8 cycles of bevacizumab, frequency of seizure decreased and follow-up MRI indicated slight reduction of peritumoral edema. During bevacizumab treatment, he suffered only transient general weakness without grade 2 side effects or above. However, his neurologic conditions had gradually worsened again. Even though additional 4 cycles of bevacizumab were given, peritumoral edema persisted (Fig. 3, 4 years of after GKRS).

**Figure 4:**
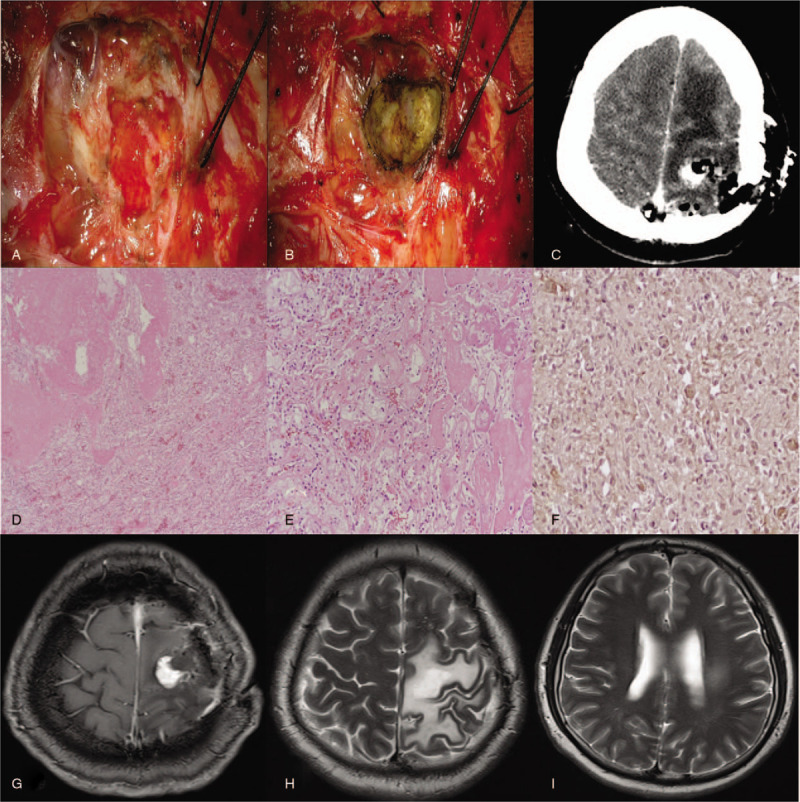
Significant adhesion to surrounding brain parenchyme with ambiguous border was observed during 2nd surgery. Radical internal debulking of tumor was achieved. (A–C) Photomicrographs showed that the tumor is composed of necrotic and viable portion that was highly vascularized, indicating angiomatous meningioma with radiation necrosis. (D and E) Immunostaning demonstrated scanty expression of VEGF. (F) At 4 months after second operation, MRI showed marked reduction of peritumoral edema (G–I).

Since no other treatment options remained, we discussed the possibility of the second operation to control intractable edema. Radical internal debulking of tumor under neurophysiologic monitoring was performed. The tumor consisted of partially necrotic and hemorrhagic portion that was severely adherent to the motor cortex. The pathological diagnosis was transformation to benign angiomatous meningioma (WHO grade I), which was previously confirmed as a meningothelial meningioma in the first operation. At 4 months after the second operation, follow-up MRI showed marked reduction of peritumoral edema (Fig. 4). He presented improvement of strength more than grade 4 in both left arm and leg, enabling ambulation without assistance. Moreover, frequency of seizure was also decreased.

## 3 Discussion

As with the glioblastoma, it is thought that VEGF is also produced in meningioma tumor cells.^[12]^ VEGF is not only localized mainly adjacent to RN but also surrounding brain parenchyme, which might lead to angiogenesis and subsequent peritumoral edema.^[5,8]^

Many studies, including a randomized double-blind placebo-controlled trial, reported that bevacizumab, a monoclonal antibody for VEGF, achieved reduction in the volume of necrosis on T2 Flair and T1-weighted gadolinium-contrast MRI with improvement in clinical symptoms.^[9,10,13]^ In previous studies, the dose of bevacizumab is usually 5 to 10 mg/kg, every 2 to 4 weeks and patients receive at least two doses (no maximum) until symptoms and imaging improved.^[14,15]^ In our case, the patient was treated for RN with bevacizumab at 5 mg/kg, q4weks, for total 12 cycles. The patient initially showed slight improvement of peritumoral edema in imaging after 8 cycles of bevacizumab. So, additional 4 cycles were used, however, the peritumoral edema was worsened again suggesting treatment failure. This failure may be related to excessive vessel reduction around RN by bevacizumab, aggravating the ischemia and hypoxia which result in paradoxically exacerbating the necrosis. Due to the bevacizumab only targets blood vessel around RN, not the necrosis itself, it can rather aggravate further necrosis.^[16]^ So, in bevacizumab-refractory cases, additional surgery is needed for accurate diagnosis and removal of necrotic core which is irreversible. In our case, the patient underwent debulking surgery for pathologic diagnosis and reducing tumor burden. The specimen showed coexistence of viable tumor tissue and partially necrotic tissue, considered as RN. A follow-up MRI at 4 months after surgery demonstrated definite reduction of peritumoral edema.

More interestingly, initial pathology demonstrated benign meningothelial meningioma, but it was transformed to benign angiomatous meningioma at 4 years after GKRS. Although, the pathogenesis is unknown, it is possible that this phenomenon is closely linked to etiology of RN. RN characterized by disruption of blood–brain barrier and vasculopathy and subsequent tissue hypoxia may trigger pathologic neovascularization resulting in angiomatous transformation.^[17]^ In addition, rich collateral veins were observed around the tumor in our case. Radiation injury causing blood vessel necrosis might result in another local hypoxia with neovascularization contributing to angiomatous transformation.

By comparing the non-angiomatous meningiomas, it is agreed that angiomatous meningiomas had higher VEGF expression and subsequently produce larger peritumoral edemas.^[18]^ So, we initially expected that extensive VEGF expression triggered by RN and angiomatous transformation may contribute to severe peritumoral edema. The specimen of 2nd surgery showed coexistence of viable tumor tissue and partially necrotic tissue, considered as RN, however, immunohistochemical staining for VEGF showed less positive reactivity than expected. This may also be related to anti-VEGF effect of bevacizumab as mentioned above.

## 4 Conclusion

This is the first report of angiomatous transformation following GKRS with pathologic confirmation. Although the pathogenesis is not clear, this rare pathologic transformation may have a close relation to RN.

Although bevacizumab has shown a good efficacy on RN, however, if bevacizumab is resistant, there are no available alternatives to treat RN. So, we suggest that debulking surgery could be an alternative treatment option to control the bevacizumab-refractory RN. Also, VEGF may not be an only contributing factor for peritumoral edema in RN.

## Author contributions

**Conceptualization:** Shin Jung

**Data curation:** You-Sub Kim, Woo-Youl Jang, Tae-Young Jung

**Supervisions:** Shin Jung, Kyung-Sub Moon

**Pathology:** Kyung-Hwa Lee

**Writing:** You-Sub Kim
